# Energy, Aromatic, and Medicinal Plants’ Potential and Prospects for the Remediation of Potentially Toxic Element-Contaminated Agricultural Soils: A Critical Meta-Analysis

**DOI:** 10.3390/toxics12120914

**Published:** 2024-12-17

**Authors:** Evangelia E. Golia, Edoardo Barbieri, Sotiria G. Papadimou, Dimitrios Alexiadis

**Affiliations:** 1 Soil Science Laboratory, School of Agriculture, Faculty of Agriculture, Forestry and Natural Environment, Aristotle University of Thessaloniki, University Campus, 54124 Thessaloniki, Greecesotiriapg@auth.gr (S.G.P.); dalexiadis@auth.gr (D.A.); 2 Faculty of Science and Technology, University of the Basque Country, 48940 Leioa, Bizkaia, Spain; 3 School of Agricultural Sciences, Department of Agriculture, Crop Production and Rural Environment, University of Thessaly, 38446 Volos, Greece

**Keywords:** soil remediation, heavy-metal-polluted soils, phytostabilization, low-cost materials, eco-friendly methods

## Abstract

A critical meta-analysis of the past decade’s investigations was carried out with the aim of assessing the use of plant-based techniques for soil remediation. Potentially toxic element (PTE) contaminated soils were selected since these contaminants are considered hazardous and have long-term effects. Furthermore, energy, aromatic, and medicinal plants were studied as their high-value products seem to be affected by PTEs’ existence. Lead (Pb), Cu, Cd, Zn, Cr, Co, Ni, Hg, and As accumulation in different parts of plant species has been investigated using proper indices. Aromatic plants seem to provide high phytoremediation yields. Increasing toxicity levels and the coexistence of many metals enhance the accumulation capacity of aromatic plants, even of toxic Cd. In plants usable as energy sources, antagonistic effects were observed, as the simultaneous presence of Cu and Cd resulted in lower thermic capacity. Finally, in most of the plants studied, it was observed that the phytostabilization technique, i.e., the accumulation of metals mainly in the roots of the plants, was often used, allowing for the aboveground part to be almost completely free of metallic pollutants. Using plants for remediation was proven to be advantageous within a circular economy model. Such a process is a promising solution, both economically and environmentally, since it provides a useful tool for keeping environmental balance and producing safe goods.

## 1. Introduction

### 1.1. Mechanisms and Methods of Phytoremediation

Phytoremediation is a technique that uses plants to remove pollutants such as PTEs from contaminated soils [1]. This method is cost-effective, environmentally friendly, and important for soil quality, preventing erosion by wind and water, and enriching soils with organic matter and microorganisms [2,3].

Different plant species have developed different mechanisms and methods to reduce the levels of potentially toxic elements (PTEs) found either in soil, water, or air [4,5,6]. For example, in phytoextraction (or phytoaccumulation), the plant extracts and thus removes the PTEs from soil [7,8]. Genetically modified species of hyperaccumulator plants have shown promising results for this mechanism [9,10]. The primary drawback of this method is its potential toxicity to pollinators, herbivores, and other animal species that come into contact with these plants [6,8,11].

In phytovolatilization, the plant transforms the PTE in the soil into one of its volatile forms and releases it into the atmosphere. PTEs such as As, Hg, and Se [12,13] can be released by different plant species as a result of the chemical transformation of inorganic to more volatile organometallic forms; however, this technique is considered controversial as large amounts of PTEs released into the atmosphere could act as a source of secondary contamination for the environment [7].

In phytostabilization, the plant makes the PTEs in the soil less bioavailable [14,15]; the main aim of phytostabilization is to decrease the soil’s metal toxicity and bioavailability to prevent metal from entering the food chain. The best way to apply phytostabilization in the field is to use native plants or combinations of different species [16,17,18].

In phytofiltration, the plant is grown in hydroponic conditions and absorbs (or adsorbs) PTE ions from the water solution in which it is growing. It can be used as an efficient way to remove PTEs such as As, Cd, and Hg [19,20,21]; however, the lack of suitable plants which can be used for this technology constitutes a major issue [22,23].

### 1.2. Plant Categories Used for Phytoremediation Purposes

Plants can absorb and adsorb metals through root and foliar systems, preventing further transport and reducing their adverse effects [4,24]. However, the main drawback of this process relies on the possibility of a future use of the contaminated biomass generated from phytoremediation [3] owing to the possibility that PTEs can enter the food chain and lead to dangerous consequences for human health [5]. For instance, using food crops such as grains, legumes, and vegetables for phytoremediation may not be environmentally safe [6,25]. Therefore, the scientific community’s interest turned to providing solutions for phytoremediation with a minor impact on the food chain and human health [5].

The cultivation of non-edible plants with high economic value in industry can be a feasible strategy to use and remediate contaminated soils with PTEs [26,27,28], allowing for environmentally safe and cost-effective phytoremediation [6]. In this regard, the use of ornamental plants for phytoremediation provides environmental and economic benefits [6,29,30]. Industrial plants can also reduce the risk of human exposure to PTEs through the food chain [26,31,32]. Utilizing plants as energy sources [12,33,34] can lead to the production of biomass for bioenergy and the fiber industry, and other valuable applications including the decontamination of soil [35,36,37]. Bioenergy production contributes to green energy production in contaminated soils in which food crops cannot grow [34,38]. Globally, plant biomass is a significant renewable energy source, and national and regional energy policies now place more emphasis on it [39,40] as it plays a progressively vital role in Europe’s energy stability [41]. The utilization of biomass energy offers a promising solution to mitigate greenhouse gas emissions and enhance environmental conditions [42]. It is projected that by 2050, bioenergy has the potential to contribute up to 15% of the world’s primary energy supply. Moreover, there is a vast expanse of approximately 1.4 billion hectares worldwide that has been identified as suitable for bioenergy production [34].

Another option is the phytoremediation of contaminated soils with medicinal and aromatic plants grown mainly for their essential oils, reducing the risk of contamination in the food chain as essential oils do not contain PTEs [6,33,43]. Growing aromatic plants for phytoremediation will facilitate restoring the soil and provide economic benefits as essential oils have great value as insect repellents and can be used in perfumery, aromatherapy, food processing, detergents, soaps, and cosmetic industries [44]. Moreover, there are no significant alterations in the essential oil composition that may impair marketability [45], and cultivation in PTE-contaminated soils may lead to an enhanced essential oil yield [25]. The most promising aromatic plant groups found for phytoremediation are Poaceae (e.g., *Arundo donax*, *Chrysopogon zizanioides*, *Cynodon dactylon*, *Panicum virgatum*, and *Phalaris arundinacea*), Asteraceae (e.g., *Cynara cardunculus* and *Silybum marianum*), and Lamiaceae (e.g., *Lavandula* spp., *Mentha* spp., *Ocimum basilicum*, *Ocimum gratissimum*, and *Rosmarinus officinalis*). These are high-value economic crops that provide financial benefits by being grown in polluted regions rather than food crops [33,44].

### 1.3. Plant Selection for Phytoremediation

**Figure 1:**
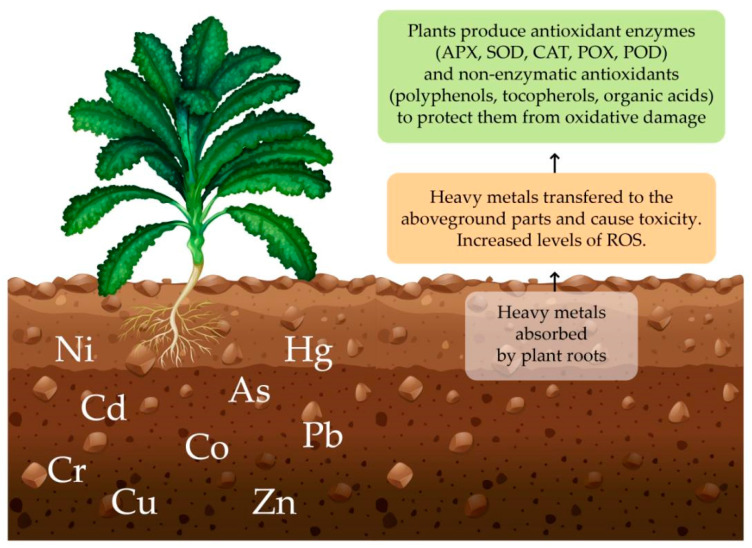
Effect of PTEs on plants.

The phytoremediation process is significantly affected by the selected plant [1,15,46] as the plants’ mechanisms of element uptake and homeostasis are highly dependent on plant species and environmental factors [47,48,49]. Generally, PTEs cause toxicity to plants, either directly or indirectly, by generating an increased quantity of reactive oxygen species (ROS). ROS, like superoxide radicals (O_2_), hydroxyl radicals (OH), and hydrogen peroxide (H_2_O_2_), are produced as byproducts associated with membrane transport activities and other metabolic pathways [50,51,52,53] (Figure 1).

Plants have antioxidant defense systems to protect them from oxidative damage caused by ROS, and detoxifying activities are very complicated and compartmentalized across plant cells [15,28,50]. Physiological, biochemical, and molecular processes play an essential role in stress tolerance, especially the antioxidant enzymes ascorbate peroxidase (APX), superoxide dismutase (SOD) [51], catalase (CAT), peroxidase (POX) [8], guaiacol peroxidase (POD), and glutathione S-transferase (GPX) [30,44], followed by non-enzymatic antioxidants, including glutathione, polyphenols, flavonoids, carotenoids, ascorbic acid, tocopherols, and organic acids [44,54].

**Figure 2:**
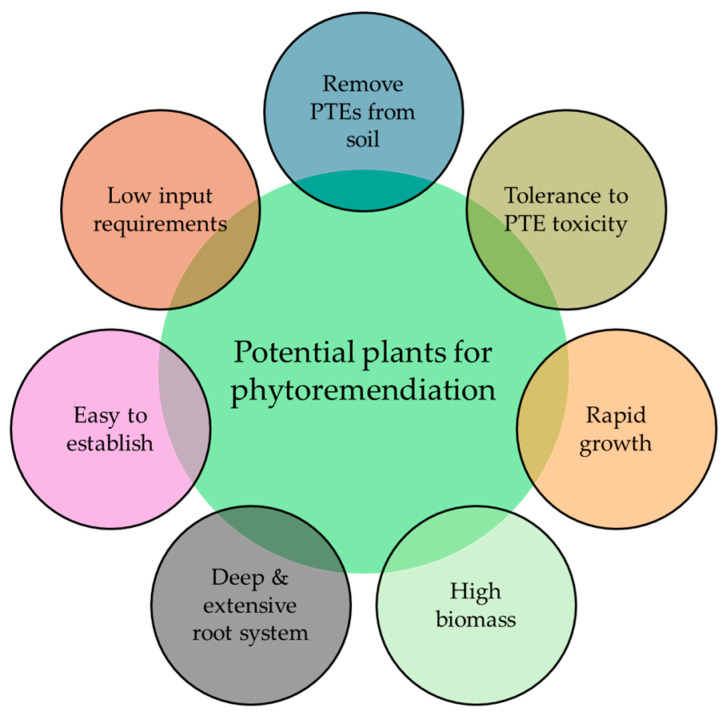
Potential plants for phytoremediation.

In this regard, phytoremediation requires plant species that are contaminant-tolerant and can also be adapted to grow in specific environmental conditions [54]. Plants are classified as indicators, accumulators, or excluders based on their function and reactivity against PTEs [44]. Plants that can take up large amounts of metals are called hyperaccumulators [55]. A common strategy for phytoremediation involves the use of hyperaccumulating plants capable of removing, stabilizing, or immobilizing PTEs in the soil [56]. However, there are restrictions in the selection of plants since numerous hyperaccumulators with a strong metal resistance and a strong acquisition of metal pollutants are much less efficient in their independent commercial operation [29]. In general, hyperaccumulating plants used for remediation can accumulate 100-fold higher metal concentrations than non-accumulator plants under the same growing conditions [57]. However, some non-accumulators can extract a comparative quantity of pollutants as hyperaccumulators owing to their greater biomass production [2]. Potential plants for phytoremediation must have the capability to remove PTEs from contaminated soil and have tolerance to PTE toxicity; they should also have rapid growth with deep and extensive root systems, produce high biomass, have excellent transpiration, and be easy to establish [2,36,58]. The suitability of the plant species also depends on crop requirements such as nutrients, water, average temperature [46], and, ideally, low input needs [38] (Figure 2). Finally, the plant species should be able to grow in the soil and climatic conditions of contaminated areas, and the use of native species is recommended to achieve optimum growth [59]. Therefore, the aim of this study was to highlight the ability of energy, aromatic, and medicinal plants to accumulate PTEs in their plant tissues and to use them for the phytoremediation of contaminated soils, reducing the risk of PTEs entering the food chain.

## 2. Materials and Methods

### 2.1. Methodology for Data Collection

**Figure 3:**
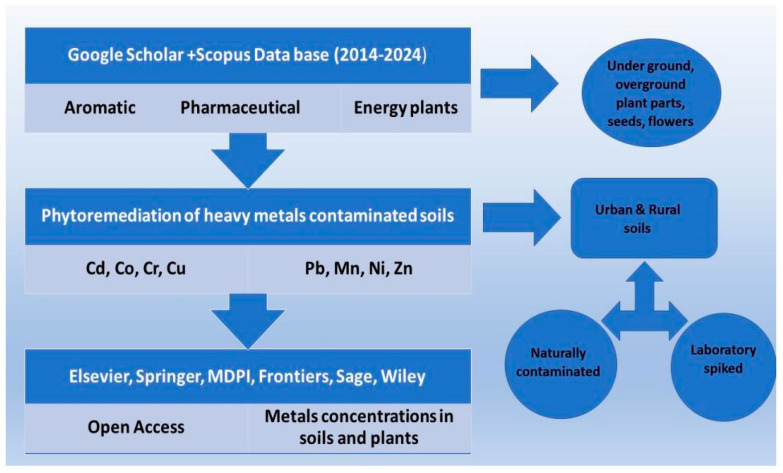
Scheme of data collection methodology-filters used to gather the published information.

In order to obtain useful data to be used in the following work, a critical meta-analysis of the available research papers regarding the phytoremediation of PTEs-polluted soils was carried out. Various online libraries and search engines, such as Google Scholar, Scopus, and ScienceDirect, were used. As a first step, papers regarding plant species suitable for phytoremediation were searched. Subsequentially, phytoremediation data for each plant species were searched on the internet using the Latin name of the plant followed by the word phytoremediation (e.g., *Silybum marianum* phytoremediation) for the period 2014–2024 (Figure 3). Therefore, the species which could give the highest amount of experimental data could be considered for this meta-analysis work. In addition to this, general information such as native country of the plant, maximum height, pH, and soil tolerance, as well as life cycle duration, were collected for each considered species. All data about PTEs absorption were standardized in mg of metal per kg of plant dry matter, and the origin of the pollution was specified (natural pollution occurring in the soil or artificial exposure to PTEs).

### 2.2. PTEs Determination

The process of establishing PTEs concentrations involves two steps: extraction using an appropriate solution or a mixture of extractants followed by the quantitative determination of the metals. Thereafter, it is necessary to identify the actual objectives of the metal quantification, and this will depend entirely on the extraction medium used. It is well known that by using diethylene triamine penta-acetic acid (DTPA) solution, the potentially available concentrations of the microelements Fe, Cu, Mn, and Zn in the plants may be determined [60]. Furthermore, it is evident that this metal concentration is proportional to the soil’s capacity to supply plants with a fraction of the total amount of metals in the soil. However, although DTPA solution extraction is widely used by soil laboratories for all soil types and for all metals, its results present analytical limitations, mainly due to the pH of the solution, which is set at 7.3 [61]. Moreover, the estimated available concentration of metals cannot be generalized for each type of plant for all climatic growing conditions as it depends on critical parameters, including the plant species, the growth stage, and the soil and climatic conditions [62].

The majority of the papers published on this topic focus on total metal concentrations, despite significant scaling and variability. For 44% of the papers reporting on total metal concentrations, extraction is achieved by using mineralization in aqua regia (a mixture of 1:3 HNO_3_:HCl), but this, explicitly, cannot extract the total metal concentration. This concentration is referred to as “pseudo-total”, which corresponds to the actual concentration, and only 16% of the works explicitly mention this term. In cases where the total amount of PTEs is to be extracted, a mixture of four strong acids (51% of the published papers referring to total metal concentration) is most often used, consisting of aqua regia, enriched with concentrated HClO_4_ (mainly to destroy the organic matter), and c. HBr (mainly to obtain the fraction of metals bound within the silicate minerals in the soil samples). A very small proportion (3%) of the published studies indicate that the solid residue is obtained after dry combustion with, e.g., HNO_3_ or, e.g., alkali solution (NaOH). Finally, a small number of published studies also point to the use of H_2_O_2_ to degrade the organic matter of soil and to recover the metals bound within it, frequently forming complex compounds (2%).

Except these cases, combinations of these have appeared as investigations have identified both available pseudo-total and total concentrations. Finally, a small category of articles (9%), refers to the determination of metal fractionation using BCR-based methods by referring to, and discriminating among, the concentration that is water-soluble and exchangeable with the soil solid surface (BCR1), the amount bound to Fe/Mn oxides (BCR2), the soil organic matter (BCR3), or the insoluble, undissolved, or residual fraction of the soil samples (BCR4) [63].

Regarding the determination of concentrations in plants, the procedure involves combustion and extraction with an acid solution, such as HCl (56% of published articles) or HNO_3_ (34%). However, there are cases where aqua regia is used [15,28]. Many of the evaluated papers note that the metals are determined in different plant parts, as Papadimou et al. [27] stated. The plant is dissected, and metals are determined separately both underground (root) and aboveground (shoots, leaves, flowers, and seeds). 

## 3. Results

### 3.1. Overview of Selected Published Studies

All plant species and studies that are included in the meta-analysis are presented in Table 1.

**Figure 4:**
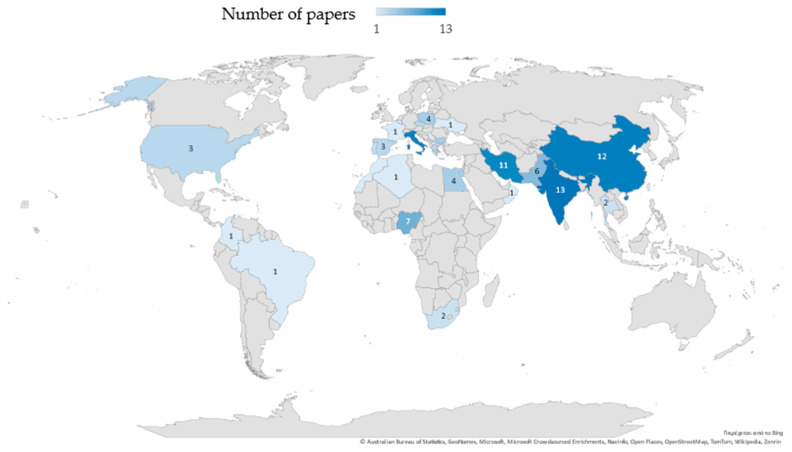
Countries where phytoremediation studies of PTEs-contaminated soils have been carried out.

In Figure 4, the number of published surveys distributed according to country are displayed. Most studies were conducted in India, followed by China, Italy, and Iran. Excluding Italy, the percentage of European countries involved ranged from 1 to 3.9%.

**Figure 5:**
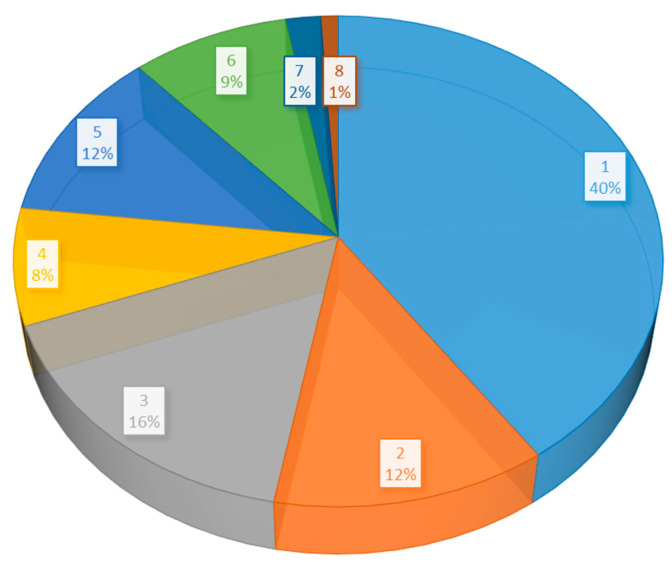
Percentages of the studies showing different metal element counts.

The majority of studies include a quantification of the highly toxic element Cd (Table 1), while a particularly high proportion of studies deal with the discrimination of metal concentrations in different parts of the plant; they determine the metal concentrations and report the results separately for the underground and aboveground parts of the plant. The percentages in the reported survey involving the phytoremediation of polluted soils with varying metal element levels are summarized in Figure 5. Only one metal has been the subject of more than 40% of surveys.

### 3.2. Pb Accumulation in Plant Parts

**Figure 6:**
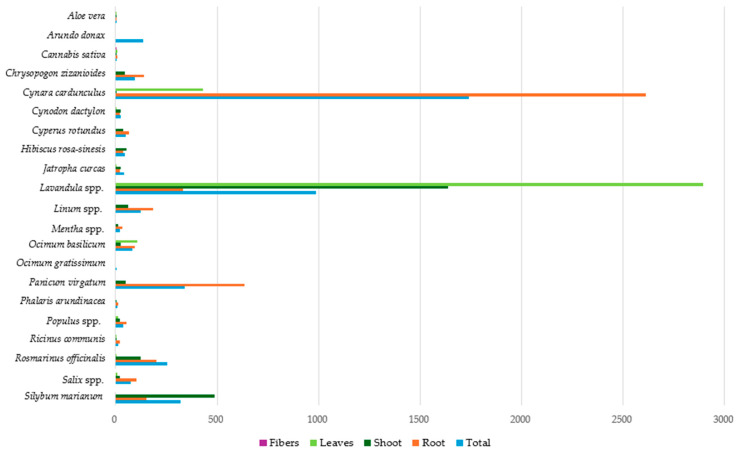
Average Pb concentration (mg kg^−1^ dw) in plant tissues.

Figure 6 presents the average Pb concentration in the different parts of the studied plants, as well as the average total Pb metal concentration per plant species. *Cynara cardunculus* appears to have the highest average total Pb concentration compared to the other species, reaching 1741.44 mg kg^−1^ (dw), followed by *Lavandula* spp., with 985.15 mg kg^−1^ (dw). On the other hand, the lowest average total Pb concentrations were observed in *Ocimum gratissimum*, *Aloe vera*, *Cannabis sativa*, and *Phalaris arundinacea* species, with 2.01, 5.75, 6.54 and 9.23 mg kg^−1^ (dw), respectively.

In the root systems of the studied plants, extremely high average Pb concentrations were identified in *Cynara cardunculus* (2613.50 mg kg^−1^ dw), while elevated concentrations were also observed in *Panicum virgatum*, *Lavandula* spp., and *Rosmarinus officinalis*, with 634.41, 331.37 and 202.10 mg kg^−1^ (dw), respectively. *Aloe vera* showed the lowest concentration, with a mean Pb concentration of 4.70 mg kg^−1^ (dw) in the root system.

By comparing the average Pb concentration in the plant shoots, the highest concentration was observed in *Lavandula* spp. (1638.93 mg kg^−1^ dw), followed by *Silybum marianum* and *Rosmarinus officinalis*, with 487.95 and 122.75 mg kg^−1^ (dw), respectively. *Cannabis sativa*, *Phalaris arundinacea*, *Aloe vera*, *Cynara cardunculus*, and *Ricinus communis* recorded the lowest mean Pb concentrations in their shoots, i.e., at less than 10 mg kg^−1^ (dw).

Finally, in plant leaves, the maximum mean Pb concentration was observed in *Lavandula* spp. (2894.65 mg kg^−1^ dw), followed by *Cynara cardunculus* and *Ocimum basilicum*, with 432.23 and 108.36 mg kg^−1^ (dw), respectively. The average Pb concentration in the leaves of all other examined plant species was below 10 mg kg^−1^ (dw), with *Cynodon dactylon* and *Ricinus communis* species exhibiting the lowest values at 0.78 and 0.38 mg kg^−1^ (dw), respectively. According to the results, *Cynara cardunculus* and *Lavandula* spp. appear to be significant Pb accumulators, while in species like *Ocimum gratissimum*, *Aloe vera*, and *Cannabis sativa*, the lowest Pb concentrations were observed.

### 3.3. Cu Accumulation in Plant Parts

**Figure 7:**
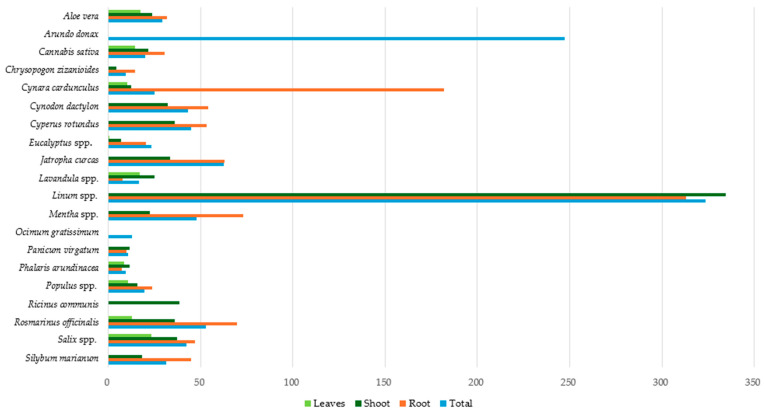
Average Cu concentration (mg kg^−1^ dw) in plant tissues.

Figure 7 presents the average Cu concentration in the different parts of the studied plants, as well as the average Cu total concentration per plant species. *Linum* spp. had the highest mean total Cu concentration among the studied species, with 323.75 mg kg^−1^ (dw), followed by *Arundo donax*, with 247.10 mg kg^−1^ (dw). In all other plant species, the average Cu total concentration was less than 60 mg kg^−1^ (dw). *Chrysopogon zizanioides* and *Phalaris arundinacea* had the lowest concentrations at 9.50 mg kg^−1^ (dw) and 9.46 mg kg^−1^ (dw), respectively.

In both the root system and shoot of *Linum* spp., exceptionally high mean Cu concentrations, i.e., exceeding 300 mg kg^−1^ (dw), were observed. An elevated average Cu concentration was also observed in the root system of *Cynara cardunculus* (181.10 mg kg^−1^ dw). The other species had concentrations below 70 mg kg^−1^ (dw), with the *Lavandula* spp. and *Phalaris arundinacea* root systems exhibiting the lowest values at 7.90 and 7.40 mg kg^−1^ (dw), respectively. The average Cu level in the shoots of all the plants tested, except for Linus spp., was less than 40 mg kg^−1^ (dw). *Eucalyptus* spp. and *Chrysopogon zizanioides* had the lowest levels, at 6.75 and 4.40 mg kg^−1^ (dw), respectively.

Finally, *Salix* spp. had the highest mean Cu concentration in leaves, with 23.36 mg kg^−1^ (dw), followed by Aloe vera and *Lavandula* spp., with 17.25 and 17.00 mg kg^−1^ (dw), respectively. *Eucalyptus* spp. leaves showed the lowest concentration, with 0.25 mg kg^−1^ (dw). According to the results, *Linum* spp. and *Arundo donax* appear to be significant Cu accumulators, while in species like *Chrysopogon zizanioides* and *Phalaris arundinacea*, the lowest Cu concentrations were observed.

### 3.4. Cd Accumulation in Plant Parts

**Figure 8:**
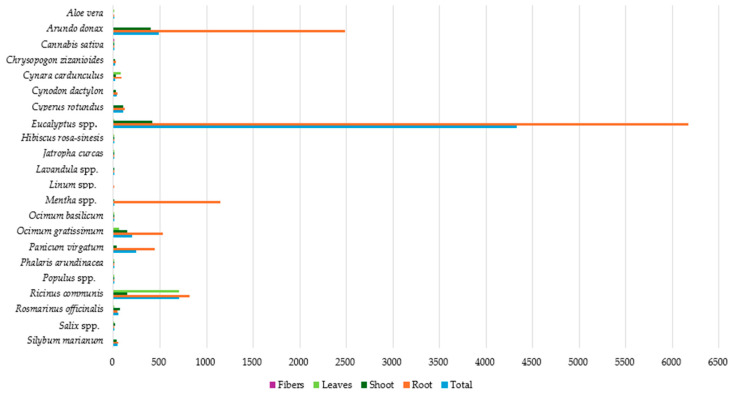
Average Cd concentration (mg kg^−1^ dw) in plant tissues.

Figure 8 presents the average Cd concentration in plant tissues, highlighting significant differences across species. Regarding the total average Cd, *Eucalyptus* spp. leads with an exceptionally high concentration (4323.59 mg kg^−1^ dw), followed by *Ricinus communis* and *Arundo donax*, with 701.50 and 486.23 mg kg^−1^ dw, respectively. On the contrary, a very low total average Cd concentration (<5 mg kg^−1^ dw) was detected in species like *Populus* spp., *Ocimum basilicum*, *Cannabis sativa*, *Hibiscus rosa-sinesis*, *Mentha* spp., *Aloe vera*, *Phalaris arundinacea*, *Lavandula* spp., and *Linum* spp.

Cd concentrations in the plant root system are similarly dominated by *Eucalyptus* spp. (6173.35 mg kg^−1^ dw), highlighting its significant Cd uptake from soil. Elevated Cd concentrations, ranging from 100 to 500 mg kg^−1^ (dw), were also detected in the roots of *Arundo donax*, *Mentha* spp., *Ricinus communis*, *Ocimum gratissimum*, *Panicum virgatum*, and *Cyperus rotundus*, while in species like *Aloe vera*, *Cannabis sativa*, *Phalaris arundinacea*, and *Lavandula* spp. Cd, mean root concentration was below 5 mg kg^−1^ (dw).

The highest shoot Cd concentration was observed in *Eucalyptus* spp., with 416.06 mg kg^−1^ dw, followed by *Arundo donax*, with 398.50 mg kg^−1^ (dw). In all other species examined, the mean Cd shoot concentration was found to be below 150 mg kg^−1^ (dw), while *Cannabis sativa*, *Mentha* spp., *Phalaris arundinacea*, and *Lavandula* spp. had the lowest mean concentrations (<1 mg kg^−1^ dw). In plant leaves, the average Cd concentration was exceptionally high in *Ricinus communis* (700 mg kg^−1^ dw).

The lowest concentrations (<1 mg kg^−1^ dw) were observed in the leaves of *Cannabis sativa*, *Rosmarinus officinalis*, and *Phalaris arundinacea.* These observations illustrate the differential ability of plants to accumulate and distribute Cd within their various tissues, which is crucial for selecting species for phytoremediation purposes. *Eucalyptus* spp. appears to possess the highest Cd concentrations, particularly in roots and shoots, indicating its strong accumulation capacity, while species like *Aloe vera*, *Phalaris arundinacea*, and *Lavandula* spp. show the lowest Cd concentrations overall, suggesting their limited Cd uptake capability.

### 3.5. Zn Accumulation in Plant Parts

**Figure 9:**
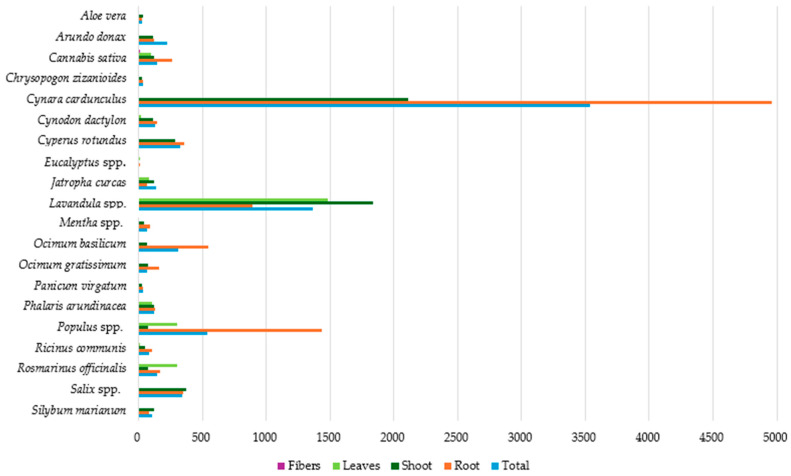
Average Zn concentration (mg kg^−1^ dw) in plant tissues.

The data in Figure 9 present the average Zn concentration (mg kg^−1^ dw) in the studied plants, revealing significant variability in Zn concentration across different species and tissues. *Cynara cardunculus* shows the highest total average Zn concentration at 3538.57 mg kg^−1^ (dw), followed by *Lavandula* spp. (1364.65 mg kg^−1^ dw) and *Populus* spp. (536.96 mg kg^−1^ dw). The lowest total average Zn concentrations (<30 mg kg^−1^ dw) were detected in *Panicum virgatum*, *Chrysopogon zizanioides*, and *Aloe vera*.

Similarly, in *Cynara cardunculus*, *Populus* spp., and *Lavandula* spp., the highest mean Zn root concentrations were observed at 4961.64, 1434.60, and 890.85 mg kg^−1^ (dw), respectively. Elevated Zn root concentrations were detected in most of the rest species studied, with the only exception being *Eucalyptus* spp.at 1.79 mg kg^−1^ dw.

The highest Zn shoot concentrations were observed once again in *Cynara cardunculus* and *Lavandula* spp. at 2115.49 and 1838.45 (mg kg^−1^ dw), respectively, while *Mentha* spp., *Aloe vera*, *Panicum virgatum*, and *Chrysopogon zizanioides* had the lowest concentrations (<40 mg kg^−1^ dw). There was a profound limitation with respect to the availability of data on Zn concentration in plant leaves. However, based on the existing data, the highest concentrations were observed in *Lavandula* spp., *Rosmarinus officinalis*, and *Populus* spp., at 1482.30, 301.00, and 299.67 mg kg^−1^ (dw), respectively, while the lowest concentration (<1 mg kg^−1^ dw) was observed in the leaves of *Eucalyptus* spp.

Therefore, *Cynara cardunculus* and *Lavandula* spp. appear to be significant Zn accumulators, whereas *Aloe vera. Panicum virgatum*, and *Chrysopogon zizanioides* were found to have minimal Zn concentrations in their tissues. A considerable variation was observed across the different species, with plant roots generally showing higher Zn concentrations compared to shoots and leaves, suggesting a higher capacity of the roots for Zn uptake and accumulation.

### 3.6. Cr Accumulation in Plant Parts

**Figure 10:**
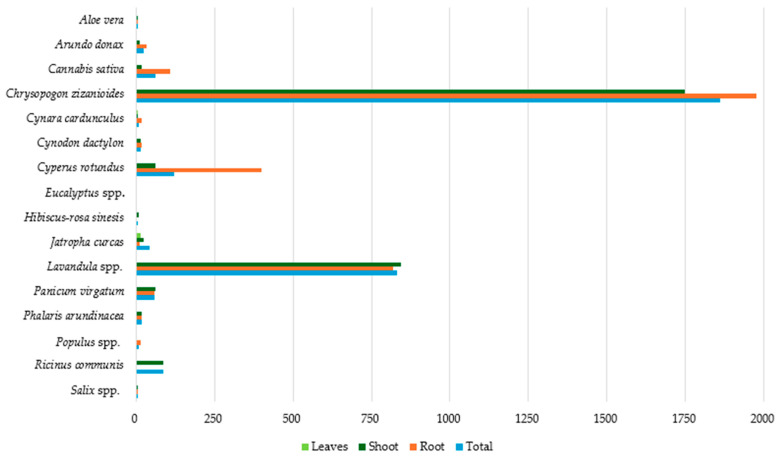
Average Cr concentration (mg kg^−1^ dw) in plant tissues.

Figure 10 presents the data on the average Cr concentration (mg kg^−1^ dw) in the studied plant species. The highest total Cr concentration was detected in *Chrysopogon zizanioides* at 1861.31 mg kg^−1^ (dw), followed by *Lavandula* spp., with a notably high concentration of 830.70 mg kg^−1^ (dw). In all other species studied, the corresponding concentrations were below 120 mg kg^−1^ (dw), with the lowest concentrations (<10 mg kg^−1^ dw) observed in *Populus* spp., *Cynara cardunculus*, *Aloe vera*, *Hibiscus-rosa sinesis*, and *Salix* spp.

High Cr concentrations were detected in the roots of *Chrysopogon zizanioides* (1974.61 mg kg^−1^ dw), *Lavandula* spp. (817.50 mg kg^−1^ dw), *Cyperus rotundus* (400.00 mg kg^−1^ dw), and *Cannabis sativa* (107.98 mg kg^−1^ dw). On the contrary, the lowest concentrations (<5 mg kg^−1^ dw) were observed in *Aloe vera*, *Salix* spp., *Hibiscus-rosa sinesis*, and *Eucalyptus* spp.

The highest Cr shoot concentrations were once again detected in *Chrysopogon zizanioides* and *Lavandula* spp., with 1748.02 and 843.90 mg kg^−1^ (dw), respectively. In the remaining species studied, Cr shoot concentration was below 85 mg kg^−1^ (dw), while the lowest was observed in *Populus* spp. (0.24 mg kg^−1^ dw).

Data availability concerning Cr concentration in plant leaves was limited. However, based on the existing data, it is noteworthy that all species were found to have low Cr concentrations (<15 mg kg^−1^ dw) in their leaves. According to the results, *Chrysopogon zizanioides* and *Lavandula* spp. appear to be significant Cr accumulators, while in species like *Salix* spp., *Hibiscus-rosa sinesis*, *Aloe vera*, *Cynara cardunculus*, and *Populus* spp., the lowest Cr concentrations were observed.

### 3.7. Ni Accumulation in Plant Parts

**Figure 11:**
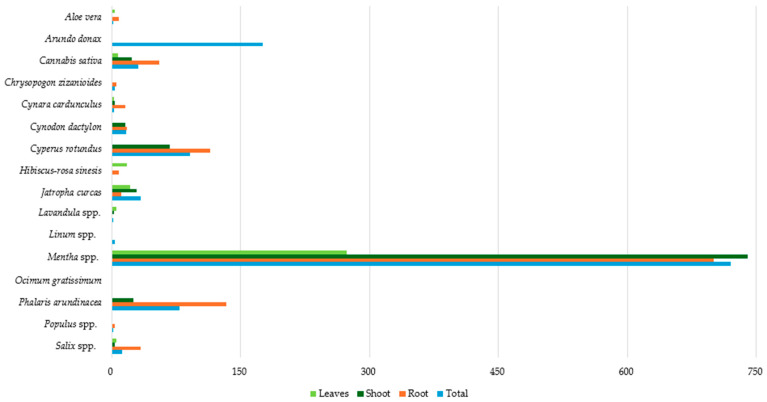
Average Ni concentration (mg kg^−1^ dw) in plant tissues.

Figure 11 presents the data on the average Ni concentration (mg kg^−1^ dw) in the studied plant species. The highest total Ni concentration was observed in *Mentha* spp. (720.09 mg kg^−1^ dw), indicating a strong ability to accumulate Ni, followed by *Arundo donax* (176.10 mg kg^−1^ dw). In contrast, the lower Ni total concentrations (<4 mg kg^−1^ dw) were detected in *Ocimum gratissimum*, *Aloe vera*, *Lavandula* spp., *Populus* spp., *Cynara cardunculus*, *Chrysopogon zizanioides*, and *Linum* spp.

Similarly, the highest root Ni concentration was found in *Mentha* spp. (700.07 mg kg^−1^ dw), while elevated concentrations were also detected in *Phalaris arundinacea* and *Cyperus rotundus* with 133.00 and 114.50 mg kg^−1^ (dw), respectively. On the other hand, the lowest root Ni concentrations (<10 mg kg^−1^ dw) were found in *Lavandula* spp., *Populus* spp., *Chrysopogon zizanioides*, *Hibiscus-rosa sinesis*, and *Aloe vera*.

Ni concentration in shoots was once again the highest in *Mentha* spp. (740.12 mg kg^−1^ dw), followed by *Cyperus rotundus* (68.00 mg kg^−1^ dw). In contrast, the lowest Ni shoot concentrations (<4 mg kg^−1^ dw) were observed in *Populus* spp., *Chrysopogon zizanioides*, *Lavandula* spp., *Salix* spp., and *Cynara cardunculus*.

Regarding Ni concentration in plant leaves for species where corresponding data were available, it was below 20 mg kg^−1^ (dw), with the only exception being *Mentha* spp. (273.57 mg kg^−1^ dw). In summary, *Mentha* spp. consistently shows high Ni concentrations in all tissues, indicating a strong capacity for Ni uptake and accumulation. On the other hand, *Aloe vera*, *Lavandula* spp., *Populus* spp., *Cynara cardunculus*, and *Chrysopogon zizanioides* had the lowest Ni concentrations for most tissues.

### 3.8. Co Accumulation in Plant Parts

**Figure 12:**
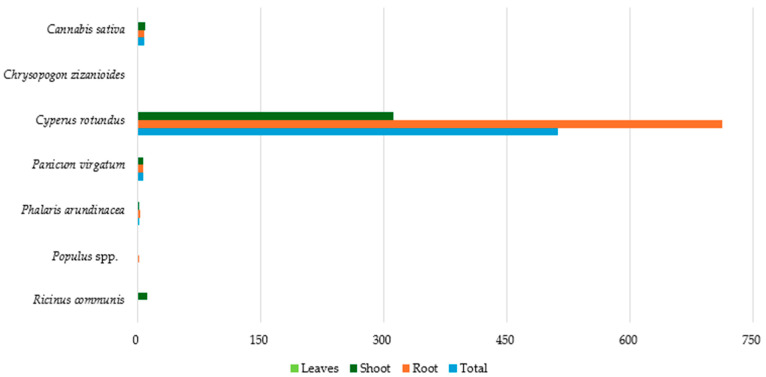
Average Co concentration (mg kg^−1^ dw) in plant tissues.

Figure 12 presents the average Co concentration in different parts of the studied plants. Data are available only for seven species (*Cyperus rotundus*, *Cannabis sativa*, *Panicum virgatum*, *Phalaris arundinacea*, *Populus* spp., *Chrysopogon zizanioides*, and *Ricinus communis*). *Cyperus rotundus* appears to have the strongest Co accumulation capacity since the concentrations detected in all its tissues were by far the highest. In all the remaining species, Co concentrations in tissues were below 12 mg kg^−1^ (dw), while the lowest (<2 mg kg^−1^ dw) were observed in *Chrysopogon zizanioides* and *Populus* spp.

### 3.9. Hg Accumulation in Plant Parts

**Figure 13:**
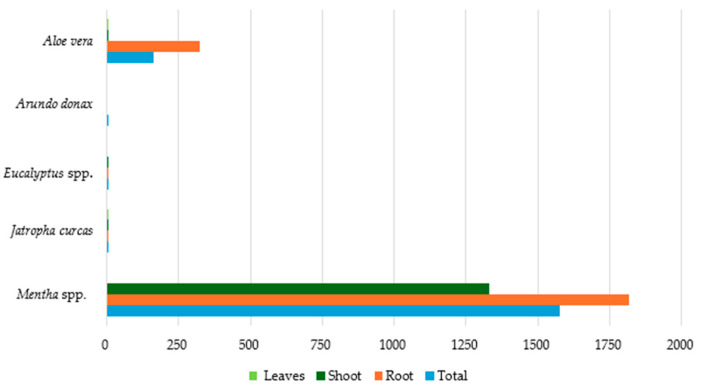
Average Hg concentration (mg kg^−1^ dw) in plant tissues.

Figure 13 presents the average Hg concentration in different parts of selected plant species (*Mentha* spp., *Aloe vera*, *Jatropha curcas*, *Arundo donax*, and *Eucalyptus* spp.) based on data availability. *Mentha* spp. was found to have the highest total Hg concentration in all its tissues, followed by *Aloe vera.* Far lower Hg concentrations (<7 mg kg^−1^ dw) were observed in the tissues of the remaining species, while the lowest were detected in all parts of *Eucalyptus* spp. (0.01 mg kg^−1^ dw).

### 3.10. As Accumulation in Plant Parts

**Figure 14:**
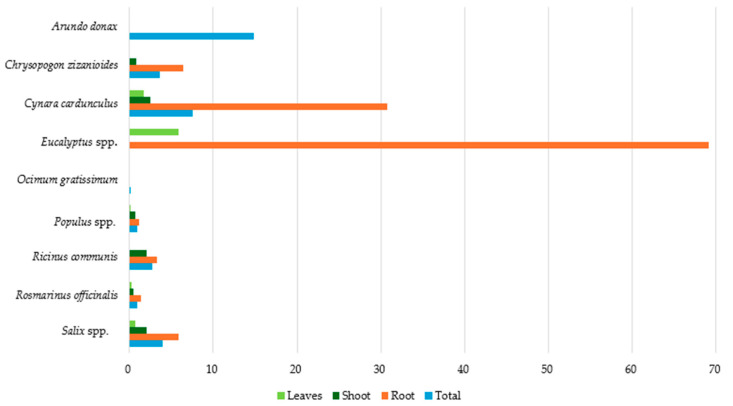
Average As concentration (mg kg^−1^ dw) in plant tissues.

Figure 14 presents the average As concentrations across different plant species and their tissues. *Arundo donax* demonstrates the highest total As concentration at 14.80 mg kg^−1^ (dw), followed by *Cynara cardunculus* at 7.50 mg kg^−1^ (dw). All the remaining species had total concentrations below 4 mg kg^−1^ (dw), whereas the lowest (<1 mg kg^−1^ dw) were detected in *Rosmarinus officinalis*, *Populus* spp., and *Ocimum gratissimum*.

In root tissues, *Eucalyptus* spp. presents a comparable high concentration of 69.17 mg kg^−1^ (dw), which is significantly higher than the other species, with *Cynara cardunculus* following at 30.76 mg kg^−1^ (dw). The lowest As root concentrations were observed in *Populus* spp. and *Rosmarinus officinalis*, with 1.11 and 1.34 mg kg^−1^ (dw), respectively.

As concentration in all plants’ shoots, based on existing data, was found to be below 2.50 mg kg^−1^ (dw). The higher shoot concentrations, ranging between 2.00 and 2.50 mg kg^−1^ (dw), were detected in *Cynara cardunculus*, *Ricinus communis*, and *Salix* spp.

Finally, considering the limited data availability, the highest As concentration in plant leaves was observed in *Eucalyptus* spp. (5.83 mg kg^−1^ dw), followed by *Cynara cardunculus* (1.70 mg kg^−1^ dw), while in the remaining species, the corresponding concentrations were found to be below 0.70 mg kg^−1^ (dw). Therefore, *Eucalyptus* spp. and *Cynara cardunculus* appear to be significant As accumulators, in contrast to *Populus* spp. and *Ocimum gratissimum*, where the lowest As concentrations were observed.

## 4. Conclusions

A critical meta-analysis of the absorption of PTEs by energy, medicinal, and aromatic plants described in the literature over the last decade was conducted to draw helpful conclusions about the possible use of these plants for phytoremediation. Most studies were conducted in Asian countries, followed by European countries, and mainly involved a single metal or a combination of metals and metalloids. In total, nine PTEs were studied, with Cd being the most extensively investigated. The accumulation and distribution of Pb, Cu, Cd, Zn, Cr, Co, Ni, Hg, and As in different parts of plant species was surveyed, concluding that the highest concentration of PTEs was observed in the plant roots. Some species seemed to be more efficient in the accumulation of certain PTEs. Specifically, *Cynara cardunculus* and *Lavandula* spp. may function as Pb accumulators; *Linum* spp. and *Arundo donax* as Cu may function as accumulators, whereas *Eucalyptus spp*. appears to have the strongest Cd accumulation; *Cynara cardunculus* and *Lavandula* spp. may function as Zn accumulatorsl *Chrysopogon zizanioides* and *Lavandula* spp. May function as significant Cr accumulator; and *Mentha* spp. may function as a strong accumulator of Ni and Hg. *Cyperus rotundus* appears to have the strongest Co accumulation capacity, while *Eucalyptus* spp. and *Cynara cardunculus* may function as arsenic accumulators.

Energy, medicinal, and aromatic plants have proved to be suitable candidates for the remediation of PTE-contaminated soils, decreasing the risk of PTE entrance to the food chain. Phytoremediation is suggested as a cost-effective and eco-friendly method, serving two distinct functions: it contributes to contaminated soil restoration, as well as to the high energy value oils, secondary metabolites, fiber, and bioenergy production, following a kind of a circular economy model.

Future work should be carried out to obtain comprehensive knowledge of the phytoremediation potential for energy, aromatic, and medicinal plants. In addition, it will serve as a guide to the utilization and management of the plant tissue, resulting from the use of phytoremediation to reduce the environmental footprint. The costs of various phytoremediation approaches should also be investigated, allowing for a comparison with other methods utilized for the remediation of PTE-contaminated soils. 

## Figures and Tables

**Table 1:** Published studies related to energy, aromatic, and medicinal plants used for phytoremediation purposes.

Plant Species	PTEs	Countries	References
*Aloe vera*	Pb, Cu, Cd, Zn, Cr, Ni, Hg	Iran, Pakistan, China	[13,52,64,65]
*Arundo donax*	Pb, Cu, Cd, Zn, Cr, Ni, Hg, As	Algeria, Portugal, Italy, India	[12,36,48,55,66]
*Cannabis sativa*	Pb, Cu, Cd, Zn, Cr, Co, Ni	India, Belgium, Poland, Italy, Croatia	[39,40,49,67,68,69,70,71,72,73]
*Chrysopogon zizanioides*	Pb, Cu, Cd, Zn, Cr, Co, Ni, As	China, Iran, Malesia, South Africa	[8,10,13,47,74]
*Cynara cardunculus*	Pb, Cu, Cd, Zn, Cr, Ni, As	Italy, Spain	[5,38,46,75]
*Cynodon dactylon*	Pb, Cu, Cd, Zn, Cr, Ni	Nigeria, India, China, Pakistan	[76,77,78,79]
*Cyperus rotundus*	Pb, Cu, Cd, Zn, Cr, Co, Ni	Nigeria, India	[76,80,81,82,83]
*Eucalyptus* spp.	Cu, Cd, Zn, Cr, Hg, As	Morocco, China, Italy, Portugal	[2,53,84,85,86]
*Hibiscus rosa-sinesis*	Pb, Cd, Cr, Ni	Egypt	[29,87]
*Jatropha curcas*	Pb, Cu, Cd, Zn, Cr, Ni, Hg	Colombia, Taiwan, Spain, Nigeria, India	[3,57,88,89,90]
*Lavandula* spp.	Pb, Cu, Cd, Zn, Cr, Ni	Bulgaria, Iran, China, Italy	[6,14,30,54,86]
*Linum* spp.	Pb, Cu, Cd, Ni	India, China, Pakistan	[1,8,55,91]
*Mentha* spp.	Pb, Cu, Cd, Zn, Ni, Hg	India, Brazil, China, Iran, Pakistan	[50,56,66,92,93]
*Ocimum basilicum*	Pb, Cd, Zn	Bulgaria, USA, Iran, Egypt	[25,45,94,95]
*Ocimum gratissimum*	Pb, Cu, Cd, Zn, Ni, As	Thailand, Nigeria, India, China	[4,43,96,97]
*Panicum virgatum*	Pb, Cu, Cd, Zn, Cr, Co	India, China, USA	[9,58,67,98]
*Phalaris arundinacea*	Pb, Cu, Cd, Zn, Cr, Co, Ni	Poland, China, Czech Republic	[37,99,100,101]
*Populus* spp.	Pb, Cu, Cd, Zn, Cr, Co, Ni, As	Egypt, Czech Republic, Pakistan, Italy	[102,103,104,105,106]
*Ricinus communis*	Pb, Cu, Cd, Zn, Cr, Co, As	Oman, Pakistan, India	[67,77,107,108,109]
*Rosmarinus officinalis*	Pb, Cu, Cd, Zn, As	Iran, France, Spain	[110,111,112,113,114]
*Salix* spp.	Pb, Cu, Cd, Zn, Cr, Ni, As	Poland, Czech Republic, Serbia, China, Egypt	[37,103,115,116,117]
*Silybum marianum*	Pb, Cu, Cd, Zn	Greece, Iran, Bulgaria, Ukraine	[15,27,118,119,120,121]

## Data Availability

Data that support the findings of this study are available from the corresponding author upon reasonable request.
